# Supplementary figures and images for: Skin microbiota variation in Indian families
Source: PeerJ. 2025 Feb 28;13:e18881. doi: 10.7717/peerj.18881 (PMC11874944; doi:10.7717/peerj.18881)

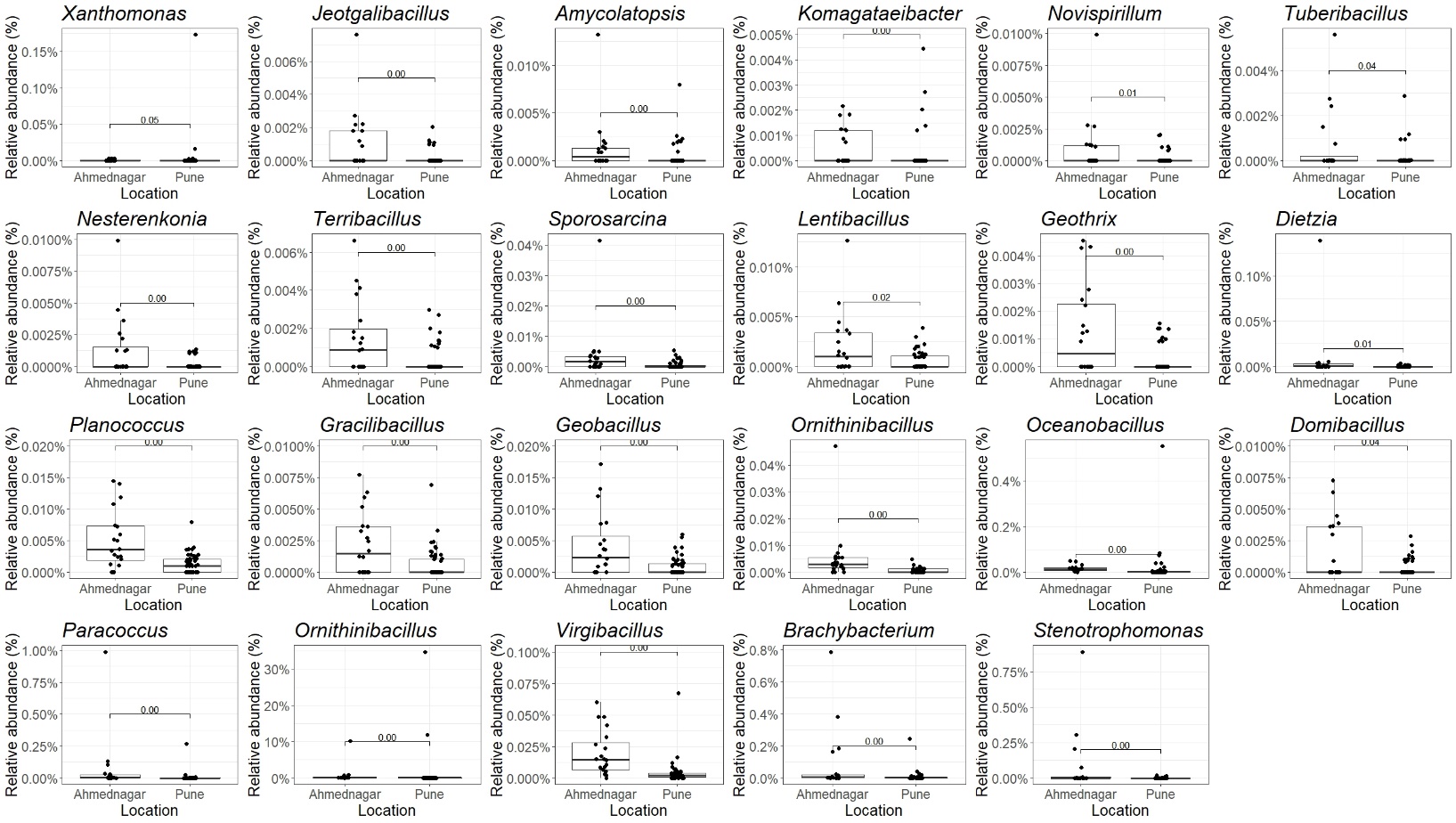

Supplement: Supplemental Information 1 — p-values were adjusted with FDR method for multiple testing for all the comparisons. [file peerj-13-18881-s001.docx]

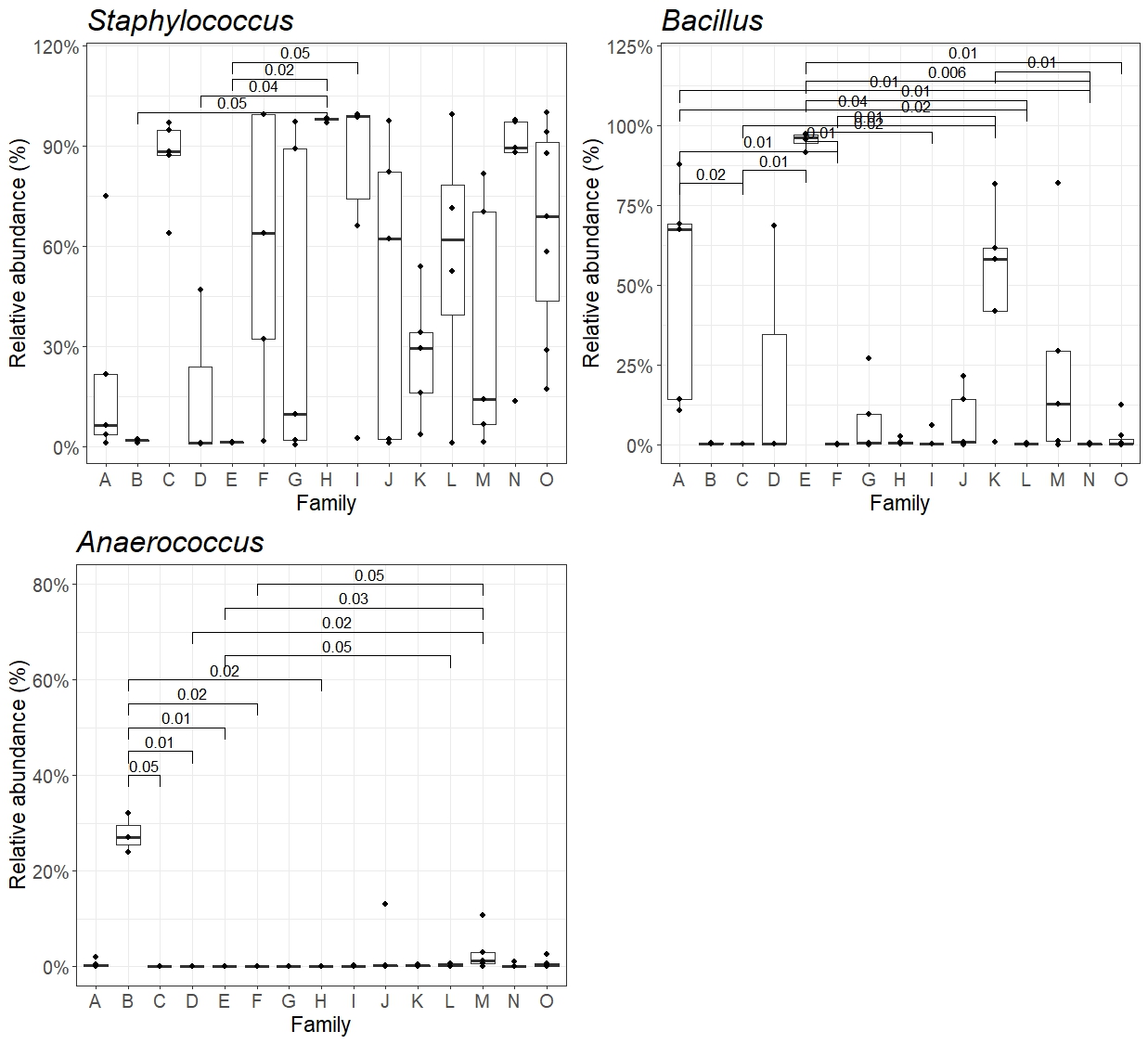

Supplement: Supplemental Information 2 — The p-values calculated based on the Dunn test using the Kruskal-Wallis test and adjusted with the “Benjamini–Hochberg (BH)” method. p-values shown only for significant family comparisons. [file peerj-13-18881-s002.docx]

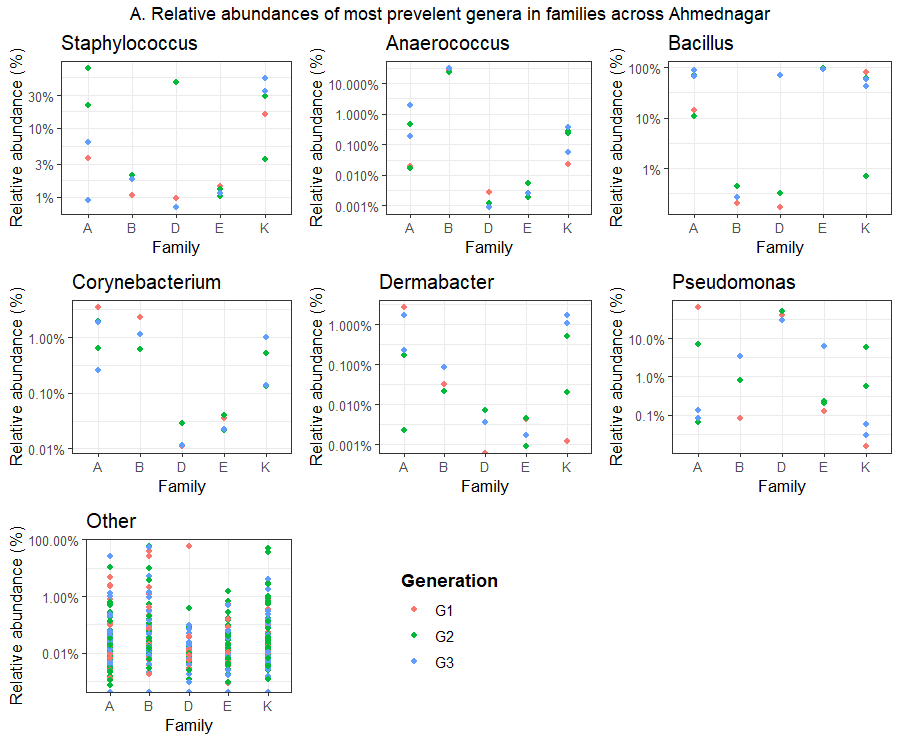


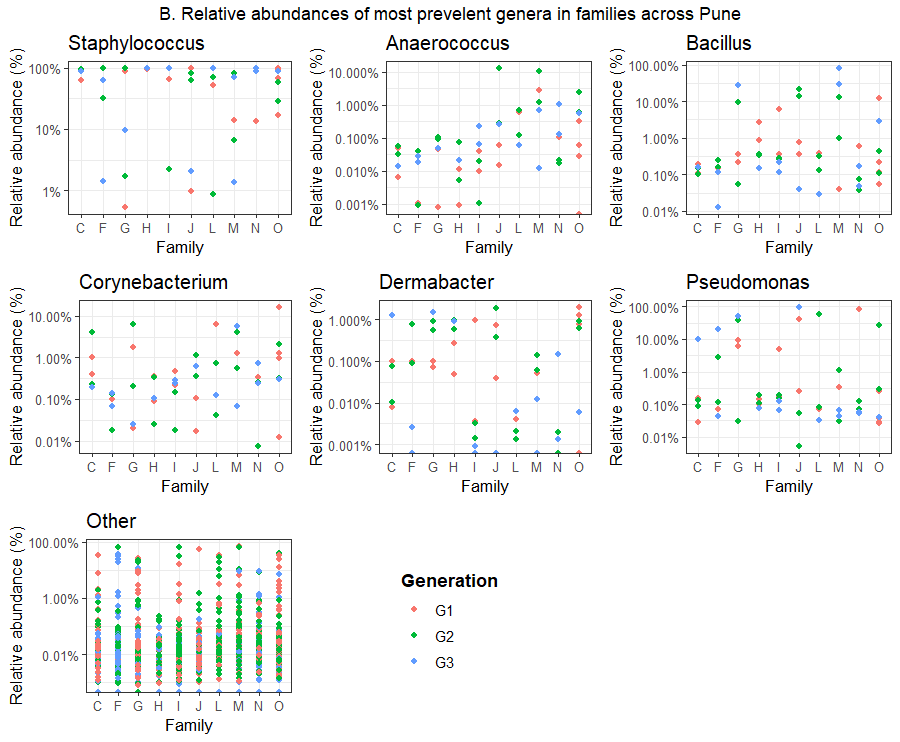

Supplement: Supplemental Information 3 — The color indicates generation (G1, G2, and G3) of the family members. [file peerj-13-18881-s003.docx]

1. Ahmednagar


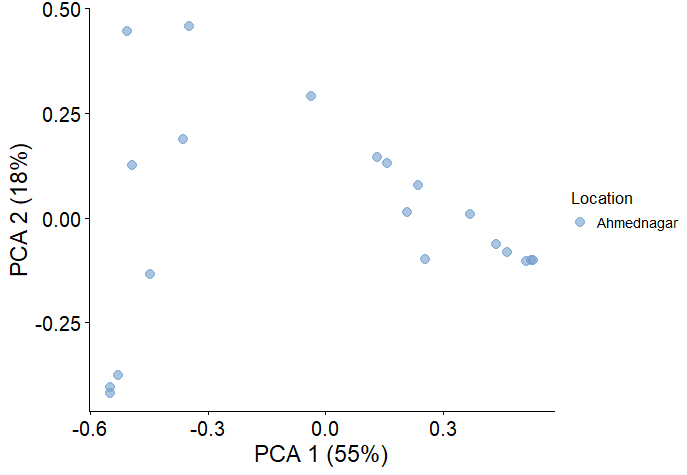


1. Pune


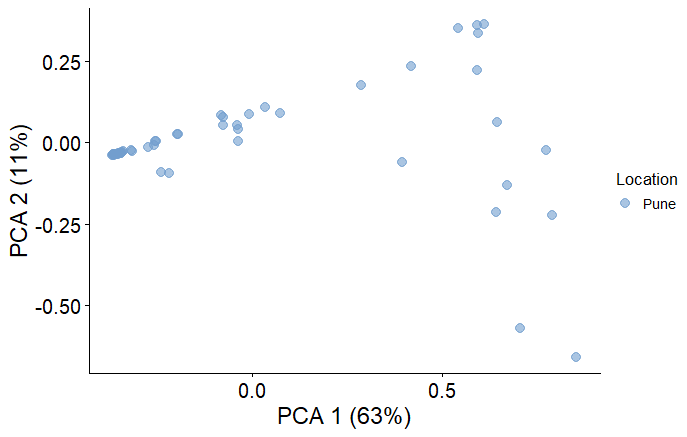

Supplement: Supplemental Information 4 — Each point represents an individual sample, color-coded by geographical location. [file peerj-13-18881-s004.docx]

A.


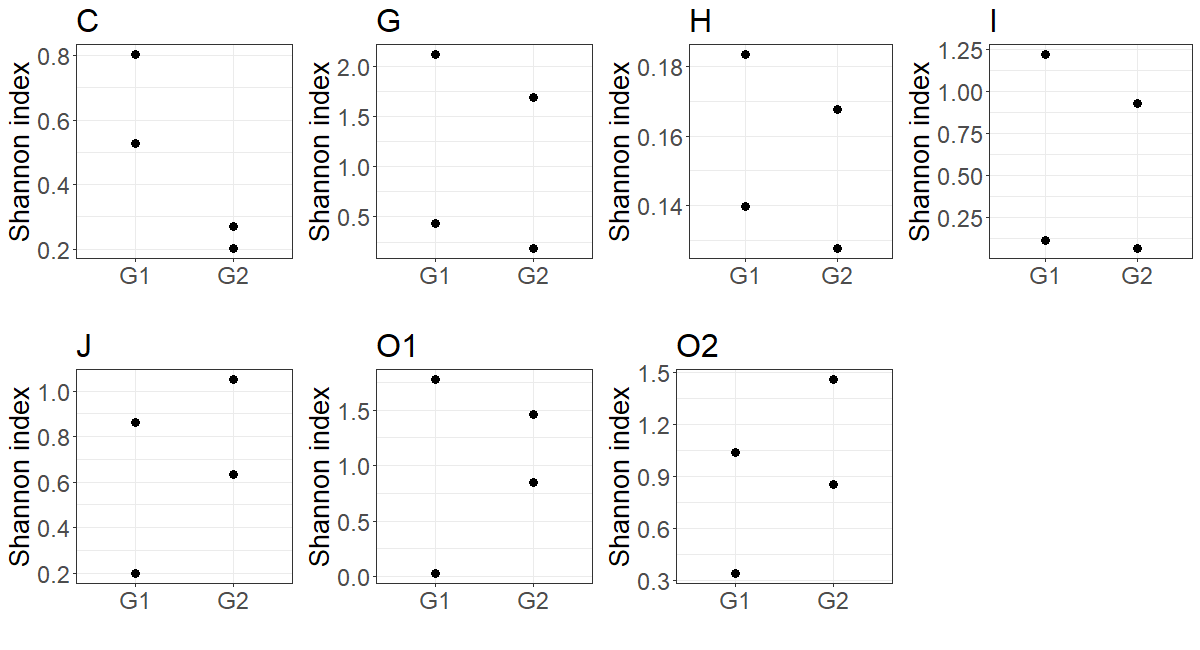

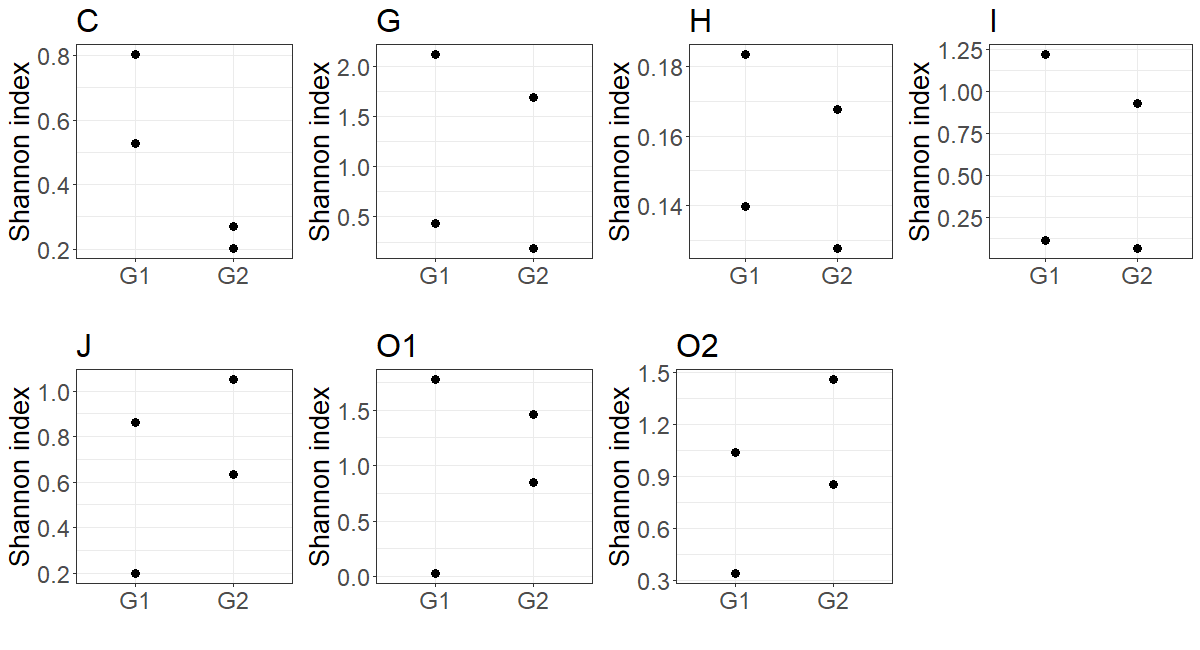


B.


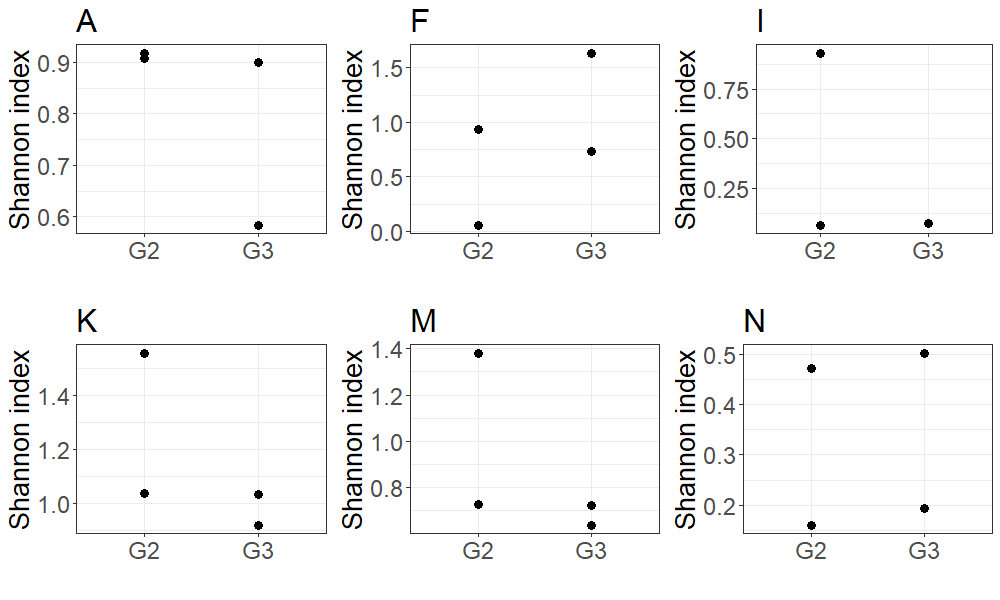

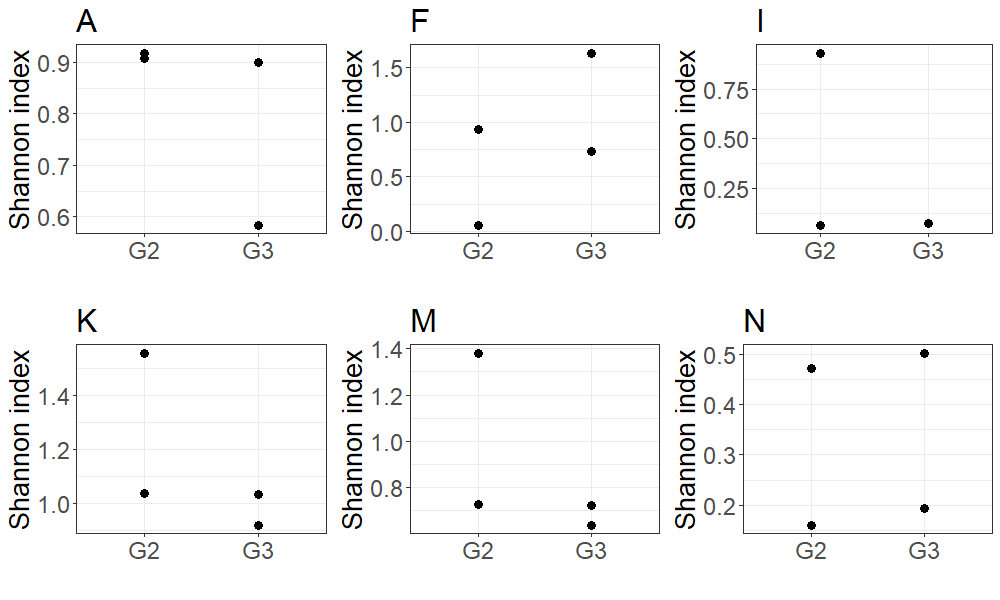

Supplement: Supplemental Information 5 — p-values were adjusted for multiple testing (Kruskal-Wallis test, FDR<0.1) for all the comparisons. [file peerj-13-18881-s005.docx]
