# Supplementary material for: Skin microbiota variation in Indian families
Source: PeerJ. 2025 Feb 28;13:e18881. doi: 10.7717/peerj.18881 (PMC11874944; doi:10.7717/peerj.18881)
Supplement: Supplemental Information 6 [file peerj-13-18881-s006.docx]

| **Sr. No.** | **Family** | **Members per family** | **Dermatological disease** | **Hypertension** | **Hypotension** | **Diabetes** | **Thyroid** | **Antibiotics** | **Physical exercise** |
| --- | --- | --- | --- | --- | --- | --- | --- | --- | --- |
| 1 | A | 5 | No | No | No | No | No | No | 2 members (40%) |
| 2 | B | 3 | No | No | No | No | No | No | 2 members (66%) |
| 3 | C | 5 | No | No | No | No | No | No | NA |
| 4 | D | 3 | No | No | No | No | No | No | NA |
| 5 | E | 4 | No | No | No | No | No | No | 2 members (50%) |
| 6 | F | 5 | No | No | No | No | No | No | 2 members (40%) |
| 7 | G | 5 | No | No | No | No | No | No | NA |
| 8 | H | 5 | No | No | No | No | No | No | 2 members (40%) |
| 9 | I | 6 | No | No | No | No | No | No | 4 members (73%) |
| 10 | J | 5 | No | No | No | No | No | No | 3 members (60%) |
| 11 | K | 5 | No | No | No | No | No | No | 3 members (60%) |
| 12 | L | 4 | No | No | No | No | No | No | NA |
| 13 | M | 5 | No | No | No | No | No | No | 3 members (60%) |
| 14 | N | 5 | No | No | No | No | No | No | NA |
| 15 | O | 7 | No | No | No | No | No | No | 4 members (56%) |
