# Supplementary material for: Skin microbiota variation in Indian families
Source: PeerJ. 2025 Feb 28;13:e18881. doi: 10.7717/peerj.18881 (PMC11874944; doi:10.7717/peerj.18881)
Supplement: Supplemental Information 7 — The p-value indicates level of significance <0.001***, <0.01**, <0.05* [file peerj-13-18881-s007.docx]

| Factor | p-value | Total variance | Explained variance |
| --- | --- | --- | --- |
| Family | 0.00*** | 5.22 | 0.42 |
| Age | 0.12 | 5.22 | 0.08 |
| Sex | 0.23 | 5.22 | 0.03 |
| Diet | NA | 5.22 | 0.00 |

Supplementary table 2 A: Ahmednagar

| Factor | p-value | Total variance | Explained variance |
| --- | --- | --- | --- |
| Family | 0.07 | 10.60 | 0.24 |
| Age | 0.69 | 10.60 | 0.02 |
| Sex | 0.83 | 10.60 | 0.00 |
| Diet | 0.89 | 10.60 | 0.00 |

Supplementary table 2 B: Pune
