# Supplementary material for: Skin microbiota variation in Indian families
Source: PeerJ. 2025 Feb 28;13:e18881. doi: 10.7717/peerj.18881 (PMC11874944; doi:10.7717/peerj.18881)
Supplement: Supplemental Information 8 [file peerj-13-18881-s008.docx]

| **Geographical location** | **G1-G3**  **within family comparison** | **Bray-Curtis dissimilarity** | **G2-G3**  **within family comparison** | **Bray-Curtis dissimilarity** | **G1-G2 within family comparison** | **Bray-Curtis dissimilarity** |
| --- | --- | --- | --- | --- | --- | --- |
| Ahmednagar | A1-A5 | 0.82 | K3-K4 | 0.96 | A1-A2 | 0.83 |
|  | D1-D3 | 0.80 | D2-D3 | 0.64 | D1-D2 | 0.75 |
|  | K1-K4 | 0.29 | A3-A5 | 0.36 | B1-B2 | 0.29 |
|  | B1-B3 | 0.20 | B2-B3 | 0.24 | K1-K2 | 0.21 |
|  | E1-E4 | 0.06 | E2-E4 | 0.06 | E1-E2 | 0.05 |
| Pune | M1-M5 | 0.96 | J3-J5 | 0.98 | G1-G5 | 0.99 |
|  | G1-G4 | 0.94 | I4-I5 | 0.98 | L1-L2 | 0.94 |
|  | N1-N4 | 0.90 | M2-M4 | 0.92 | N1-N2 | 0.87 |
|  | F1-F4 | 0.64 | F3-F4 | 0.66 | F1-F2 | 0.68 |
|  | J2-J5 | 0.42 | O6-O7 | 0.64 | I1-I3 | 0.50 |
|  | L1-L4 | 0.37 | G3-G4 | 0.49 | M1-M2 | 0.41 |
|  | H2-H5 | 0.28 | N2-N4 | 0.28 | O1-O5 | 0.31 |
|  | C1-C5 | 0.27 | H3-H5 | 0.23 | J1-J3 | 0.23 |
|  | O1-O7 | 0.26 | L3-L4 | 0.22 | C1-C3 | 0.23 |
|  | I2-I6 | 0.15 | C4-C5 | 0.14 | H1-H3 | 0.07 |
