# Supplementary material for: Skin microbiota variation in Indian families
Source: PeerJ. 2025 Feb 28;13:e18881. doi: 10.7717/peerj.18881 (PMC11874944; doi:10.7717/peerj.18881)
Supplement: Supplemental Information 9 — The Bray-Curtis values are arranged in decreasing order within the geographical location. [file peerj-13-18881-s009.docx]

| **Geographical location** | **G2-G2**  **within family comparison** | **Bray-Curtis dissimilarity** |
| --- | --- | --- |
| Ahmednagar | K2-K3 | 0.97 |
|  | A2-A3 | 0.69 |
|  | E2-E3 | 0.03 |
| Pune | I3-I4 | 0.97 |
|  | G3-G5 | 0.97 |
|  | M2-M3 | 0.91 |
|  | L2-L3 | 0.83 |
|  | F2-F3 | 0.70 |
|  | N3-N4 | 0.43 |
|  | C2-C3 | 0.34 |
|  | O5-O6 | 0.32 |
|  | J3-J4 | 0.19 |
|  | H3-H4 | 0.02 |
